# Comparative effectiveness of pemafibrate versus bezafibrate on hepatic and vascular endothelial function in patients with coronary artery disease and metabolic dysfunction-associated steatotic liver disease

**DOI:** 10.3389/fendo.2026.1750308

**Published:** 2026-02-11

**Authors:** Akihiro Nakamura, Yuta Kagaya, Hiroki Saito, Masanori Kanazawa, Masanobu Miura, Masateru Kondo, Hideaki Endo

**Affiliations:** Department of Cardiology, Iwate Prefectural Central Hospital, Morioka, Japan

**Keywords:** alanine aminotransferase, fatty liver index, flow-mediated vasodilation, homeostasis model assessment of insulin resistance, peroxisome proliferator-activated receptor-α

## Abstract

**Background:**

Peroxisome proliferator-activated receptor (PPAR) agonists are promising therapeutic agents for metabolic dysfunction-associated steatotic liver disease (MASLD), which increases cardiovascular risk.

**Methods:**

We compared the efficacy of pemafibrate, a highly selective PPAR-α agonist, and bezafibrate, a non-selective PPAR-α agonist, in patients with coronary artery disease (CAD) and MASLD. This was a *post-hoc* analysis of a randomized, crossover study that treated hypertriglyceridemia with pemafibrate or bezafibrate for 24 weeks, followed by a crossover for another 24 weeks. Of the 60 enrolled patients, 21 were identified as having MASLD (fatty liver index [FLI] ≥ 60 and hepatic steatosis index [HSI] ≥ 36 as indicators). The primary outcomes were changes in serum alanine aminotransferase (ALT) levels and flow-mediated dilation (FMD). Secondary outcomes were changes in the FLI and HSI. Homeostasis model assessment of insulin resistance (HOMA-IR) was assessed as an exploratory outcome.

**Results:**

The percentage reduction in ALT levels was significantly greater with pemafibrate treatment than with bezafibrate treatment (−23.1% vs. −9.2%, P = 0.035). FMD significantly increased in both groups, with no difference in the magnitude of the percentage change (P = 0.267). FLI significantly decreased in both groups with no difference in the magnitude of change (P = 0.983), while HSI was not significantly different before and after treatment in either group. Both treatments significantly lowered HOMA-IR; however, the decrease was similar between the groups (P = 0.724).

**Conclusions:**

Pemafibrate is more effective than bezafibrate at reducing ALT levels while offering similar beneficial effects on insulin resistance and endothelial function in CAD patients with MASLD.

## Introduction

Metabolic dysfunction-associated steatotic liver disease (MASLD), previously known as non-alcoholic fatty liver disease (NAFLD), is the most prevalent chronic liver condition, affecting approximately a quarter of the global adult population (1, 2). Since MASLD is often associated with metabolic disorders such as type 2 diabetes mellitus (DM) and dyslipidemia (3), it can significantly increase the risk of coronary artery disease (CAD) (4). Therefore, treating MASLD in patients with CAD is crucial not only for reducing the risk of developing CAD but also for preventing the progression of liver-related complications such as cirrhosis and hepatocellular carcinoma (5).

It has been demonstrated that disturbances in the lipid metabolic balance in the liver lead to lipid accumulation and consequently, hepatocellular toxicity and MASLD (5). Because peroxisome proliferator-activated receptors (PPARs) play a crucial role in transcriptional regulation as modulators of lipid and glucose metabolism, fibrates, which are PPAR agonists, have been investigated as potential therapeutic agents for MASLD. In particular, pemafibrate, a highly selective PPAR-α modulator, has shown efficacy in patients with MASLD by lowering triglyceride and liver enzymes levels (6). However, the effectiveness of pemafibrate and other existing fibrates in patients with MASLD has not been adequately compared. Bezafibrate was developed as an agonist for all three isoforms of human PPAR (PPAR-α, PPAR-γ, and PPAR-δ), and has been widely used both in Japan and internationally (7, 8). Previously, we examined the efficacy and safety of pemafibrate and bezafibrate on lipid profile and liver and renal function using a randomized crossover method (9). In this *post-hoc* analysis, we compared the effects of pemafibrate and bezafibrate on liver enzyme levels in patients with statin-treated CAD and suspected MASLD. Furthermore, we investigated the impact of pemafibrate and bezafibrate on flow-mediated vasodilation (FMD) to assess endothelial function in these patients, because MASLD has been reported to be significantly associated with endothelial dysfunction compared to non-MASLD patients (10). Despite standard pharmacological interventions, patients with comorbid CAD and MASLD remain at high risk for recurrent cardiovascular events. Investigating additional therapies to further improve endothelial function in this population is crucial for addressing the unmet medical need of residual vascular risk.

## Methods

### Study design and patient population

This study used data from patients who participated in the Pemafibrate and Bezafibrate (PEBE) study. The PEBE study was a single-center, prospective, randomized, open-label, crossover study designed to determine and compare the efficacy and safety of pemafibrate and bezafibrate treatments in patients with statin-treated CAD and dyslipidemia. Detailed information about the patients and the PEBE study protocol has been published previously (9). Briefly, 60 patients aged 20–75 years, diagnosed with CAD and dyslipidemia and undergoing statin therapy (with a fasting serum TG level of ≥ 150 mg/dL and HDL-C level of < 50 mg/dL in men or < 55 mg/dL in women at the start of the study) were administered pemafibrate 0.2 mg/day or bezafibrate 400 mg/day for 24 weeks (Period 1: pre-washout treatment period), followed by crossover to the alternate medication for another 24 weeks (Period 2: post-washout treatment period). A 4-week washout period was implemented between the two treatment periods, during which the participants did not receive pemafibrate or bezafibrate. Laboratory tests and FMD measurements were performed before and after each 24-week treatment. All concomitant medications and lifestyle interventions (diet and exercise) were kept constant during the entire study period to avoid confounding the effects of the study drugs. The PEBE study was registered in the University Hospital Medical Information Network Clinical Trials Registry (UMIN000047737).

**Figure 1:**
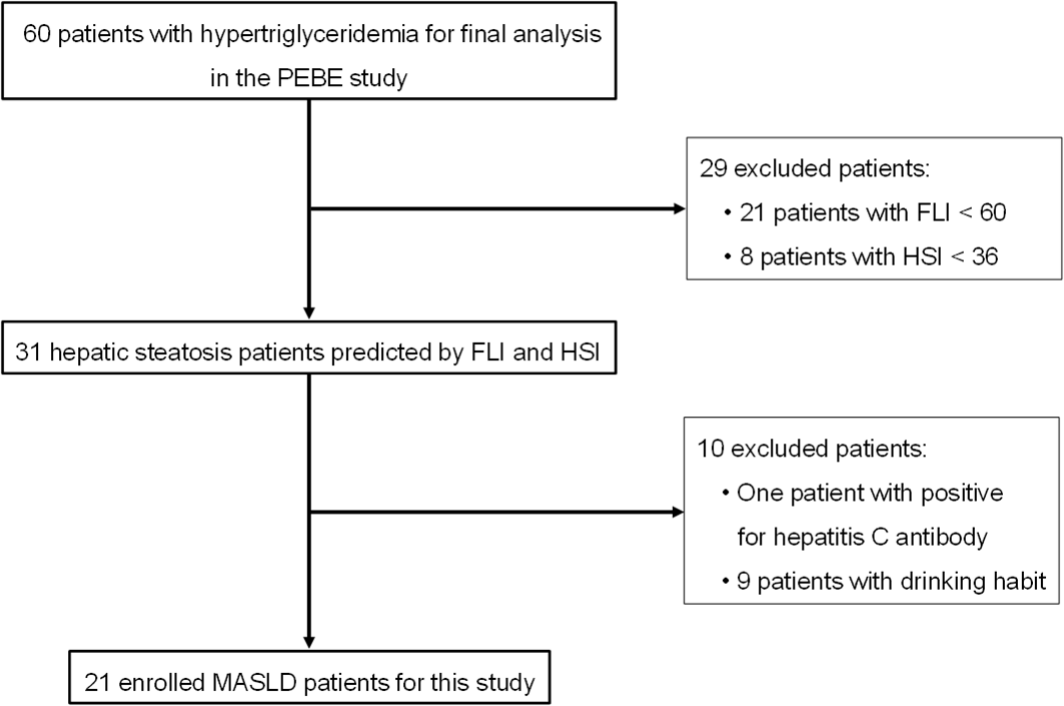
Flowchart of patient selection. The PEBE study indicates the comparative study of pemafibrate and bezafibrate. FLI, fatty liver index; HSI, hepatic steatosis index; and MASLD, metabolic dysfunction-associated steatotic liver disease.

A flow chart of the study patients with MASLD is shown in **Figure 1**. The main exclusion criteria were as follows: patients positive for hepatitis B surface antigen or hepatitis C antibody, patients with excessive alcohol consumption (alcohol equivalent: ≥ 30 g/day in men; ≥ 20 g/day in women); and patients with a fatty liver index (FLI) < 60 and hepatic steatosis index (HSI) < 36 (11). After applying the prespecified exclusion criteria, a total of 21 patients (men/women: 20/1; mean age: 61 ± 9 years) were identified as having MASLD (FLI ≥ 60 and HSI ≥ 36) and enrolled in the study. That is, patients with CAD and non-alcoholic hepatic steatosis, as indicated by non-invasive biomarkers, were diagnosed with MASLD based on hypertriglyceridemia, a key risk factor for MASLD (1). In this *post-hoc* analysis, data were extracted from the database of the PEBE study (9) and analyzed as a single cohort, following the original crossover protocol. The 4-week washout period was applied as predetermined in the primary study. To maintain consistency with the published findings in the PEBE study (9), the treatment effects were evaluated across the total participant group.

This study was conducted in accordance with the Declaration of Helsinki. Written informed consent was obtained from all patients. The study protocol was approved by the Ethics Committee of Iwate Prefectural Central Hospital (approval no. 2738).

### Definitions and measurements of biochemical markers

FLI and HSI were used in this study for the diagnosis of suspected MASLD (11, 12). These scores were developed as non-invasive, non-imaging predictors of MASLD (1) and were calculated based on typical risk factors for the disease, including body mass index (BMI), waist circumference (WC), and serum levels of γ-glutamyltransferase (γGT), triglycerides, and liver transaminases (13, 14). The optimal cutoff score for FLI was set at ≥ 60, with a corresponding sensitivity of 61%, specificity of 86%, and accuracy of 84%, as determined by ultrasonography (13, 15). The HSI is a widely used hepatic steatosis score, especially in Asian populations, with an optimal cutoff score for detecting suspected MASLD set at 36 (16). Therefore, FLI ≥ 60 and HSI ≥ 36 was used as indicators of hepatic steatosis in this study. The formula for FLI was as follows: FLI = [e (0.953 × Log_e_ (triglyceride + 0.139 × BMI + 0.718 × Log_e_ (γGT) + 0.053 × WC –15.745)/[1 + e (0.953 × Log_e_(triglyceride) + 0.139 × BMI + 0.718 × Log_e_ (γGT) + 0.053 × WC –15.745)] × 100 (15). The formula for HSI was as follows: HSI = 8 × [alanine aminotransferase (ALT, U/L)/aspartate aminotransferase (AST, U/L)] + BMI + 2 (if type 2 DM was present) + 2 (if the patient was a woman) (16). BMI was calculated by dividing the weight in kilograms by the square of the height in meters, and WC was measured midway between the lowest rib and the iliac crest in a standing position after a regular exhalation. In this study, the Fibrosis-4 (FIB-4) index, a useful non-invasive marker for evaluating liver fibrosis including MASLD, was also calculated using the following formula: FIB-4 = [age (years) × AST (U/L)]/[platelet count (10^9^/L) × ALT (U/L)^1/2^] (17).

Hypertension was diagnosed if the systolic and/or diastolic blood pressure (BP) was ≥ 140/90 mmHg or if antihypertensive medications were used. DM was diagnosed according to the American Diabetes Association criteria which include: (1) a fasting plasma glucose (FPG) level of ≥ 126 mg/dL, (2) a glycated hemoglobin A1c (HbA1c) level of ≥ 6.5%, or the current use of hypoglycemic agents (18).

The following serum marker levels were measured in a commercial laboratory (SRL Inc., Tokyo, Japan): triglyceride (mg/dL), low-density lipoprotein cholesterol (LDL-C, mg/dL), high-density lipoprotein cholesterol (HDL-C, mg/dL), lipoprotein (a) (Lp(a), mg/dL), remnant lipoprotein cholesterol (RemL-C, mg/dL), apolipoprotein A-I (Apo A-I, mg/dL), apolipoprotein B (Apo B, mg/dL), apolipoprotein B-48 (Apo B-48, μg/dL), AST (U/L), ALT (U/L), γ-GT (U/L), HbA1c (%), creatinine (mg/dL), cystatin C (mg/L), and creatine kinase (CK, U/L), as previously reported (9). The estimated glomerular filtration rate (eGFR) was calculated based on the serum creatinine and cystatin C levels (19, 20).

Patients with stable CAD were defined as those with cardiac ischemia who had a history of myocardial infarction, coronary artery bypass, percutaneous coronary intervention with or without stenting, or a previously angiographically proven stenotic lesion of ≥ 75% in a major epicardial coronary artery. A stable condition was defined as chest pain induced by exertion, which was relieved by nitrate therapy, and which did not change in its characteristics (frequency, severity, duration, time of appearance, and precipitating factors) for the 12 weeks preceding the study.

### Assessment of insulin resistance

To assess insulin resistance, we examined changes in the following markers before and after 24 weeks of treatment: (1) serum insulin levels and (2) homeostasis model assessment of insulin resistance (HOMA-IR), which was calculated as FPG (mg/dL) × fasting plasma insulin (μIU/mL)/405. Plasma glucose (mg/dL) and serum insulin (μIU/mL) levels were measured in the laboratory.

### Measurement of FMD

FMD was measured to assess endothelium-dependent vascular function in the brachial artery. Two trained ultrasonographers blinded to the study details measured brachial FMDs using a semi-automated edge detection system (UNEXEF18G equipment; UNEX, Nagoya, Japan), following the guidelines (21). Briefly, the right brachial artery was scanned using high-resolution ultrasound with a 10 MHz linear array transducer to obtain longitudinal and transverse images while the patient was in a supine position after resting for ≥ 15 min. A sphygmomanometric cuff connected to UNEXEF18G was placed around the right forearm, and images of the artery were captured at the proximal portion of the antecubital fossa. After recording the baseline images, the cuff was inflated to at least 50 mmHg above the systolic BP for 5 min and then deflated. The post-deflation arterial images were captured in a manner similar to that for reactive hyperemia, and artery diameters were measured for 2 min using R-wave synchronized automated edge- detection software. Brachial artery FMD was calculated as the maximum post-deflation diameter relative to the average baseline diameter. To minimize the influence on FMD measurement, all nitrates were withheld for at least 24 hours before the test, and the use of any sublingual nitrates was prohibited on the morning of the examination. We assessed inter- and intra-reader variability in 100 randomly selected and blinded FMD scan images. We found no statistically significant differences between the first and second %FMD measurements for both observers (–0.13%, 95% CI−1.07 to 0.75%; 0.07%, 95% CI−0.86 to 0.61%) or between the observers (–0.09%, 95% CI−0.74 to 0.59%). The repeatability of the measurement between the two observers was high (r = 0.89).

### Study outcomes

The primary outcomes were the changes or percentage changes in serum ALT levels and FMD before and after 24 weeks of treatment with pemafibrate compared to treatment with bezafibrate. The percentage change was calculated as follows: ([value after treatment − value before treatment]/value before treatment × 100.

The secondary outcomes were assessed in the pemafibrate treatment group and compared with those in the bezafibrate group. The changes or percentage changes before and after the treatment were evaluated for the following: (1) serum AST and γGT levels and (2) FLI and HSI scores.

Other exploratory outcomes were also assessed, including the percentage changes before and after treatment for insulin resistance-related markers, such as FPG, fasting serum insulin, and HOMA-IR.

### Statistical analyses

All values are presented as the mean ± standard deviation for continuous variables and as numbers and percentages for categorical variables. Continuous variables were evaluated for normal distribution using the Kolmogorov–Smirnov test. Differences in the parameters between the pemafibrate and bezafibrate treatments were assessed with Student’s t-tests if the distributions in both treatments were normal, and with Mann–Whitney U-tests otherwise. Within-group changes in the parameters were evaluated with paired t-tests if the distributions of the variables were normal and with Wilcoxon signed-rank tests otherwise. Differences in categorical variables between pemafibrate and bezafibrate treatments were assessed using the chi-square test or Fisher’s exact test, as appropriate. Statistical significance was set at P < 0.05. All statistical analyses were performed using Excel (Microsoft, Redmond, WA, USA) with the Statcel4 add-in software.

## Results

From the original cohort of 60 participants, 21 patients with MASLD were retrospectively identified for this *post-hoc* analysis. In Period 1, 9 patients received pemafibrate and 12 received bezafibrate; these treatments were crossed over in Period 2. We compared the efficacy of pemafibrate versus bezafibrate by pooling data from both periods. The pooled results are described below, while individual data for each period are provided in the **Supplementary Materials** (**Supplementary Tables 1**-**5**).

### Patient characteristics

The pretreatment clinical characteristics of patients with MASLD (n = 21) are summarized in **Table 1**. The mean age was 61 years, and the majority of patients (20/21, 95%) were men. Approximately half of the patients (n = 11, 52%) had type 2 DM and 86% (n = 18) had hypertension. The mean BMI, FLI, and HSI were 28.4 kg/m^2^, 80.4, and 36.8, respectively. The mean FIB-4 index was 1.48 (< 1.3: low risk for advanced liver fibrosis, n = 9; 1.3–2.67: intermediate risk, n = 11; > 2.67: high risk, n = 1).

**Table 1:** Patient characteristics.

Characteristics	(n = 21)
Age (years)	61 ± 9
Sex, men	20 (95)
WC (cm)	99.0 ± 7.4
Body weight (kg)	81.6 ± 12.5
BMI (kg/m^2^)	28.4 ± 2.9
Current or former smoker	14 (67)
Hypertension	18 (86)
Type 2 DM	11 (52)
Fasting plasma glucose (mg/dL)	123.5 ± 40.4
HbA1c (%)	6.3 ± 0.9
Systolic BP (mmHg)	131.5 ± 26.2
Diastolic BP (mmHg)	82.0 ± 12.8
Platelet count (10^4^/μL)	23.1 ± 6.1
FLI	80.4 ± 11.5
HSI	36.8 ± 2.8
FIB-4 index	1.48 ± 0.59
Medication	
Statins: Rosuvastatin/Pitavastatin/Atorvastatin	21 (100): 10 (48)/7 (33)/4 (19)
RAS inhibitors (ACEi/ARB)	15 (71)
SGLT2 inhibitors	3 (14)
Metformin	5 (24)
Calcium channel blockers	11 (52)
History of PCI with DES	13 (62)

Variables are expressed as mean ± SD or number (%). WC, waist circumference; BMI, body mass index; DM, diabetes mellitus; HbA1c, glycated hemoglobin A1c; BP, blood pressure; FLI, fatty liver index; HSI, hepatic steatosis index; and FIB-4, fibrosis-4; RAS, renin-angiotensin system; ACEi, angiotensin-converting enzyme inhibitor; ARB, angiotensin receptor blocker; SGLT2, sodium glucose co-transporter 2; PCI, percutaneous coronary intervention; DES, drug-eluting stent.

### Physiological, lipid markers, and liver enzymes before and after pemafibrate or bezafibrate treatment

**Table 2** shows the percentage changes in physiological and lipid markers before and after treatment with pemafibrate or bezafibrate. No significant differences in body weight, BMI, or WC were observed between the treatment groups. Serum triglyceride levels decreased significantly with both treatments, and the percentage changes were significantly greater with pemafibrate treatment than with bezafibrate treatment (P = 0.017). The results for other lipid parameters showed significant increases in serum HDL-C and Apo A-I levels, as well as significant decreases in serum Apo B, RemL-C, and Apo B-48 levels in both treatment groups. The percentage changes in serum RemL-C and Apo B-48 levels were significantly greater with pemafibrate treatment than with bezafibrate treatment (RemL-C, P = 0.030; Apo B-48, P = 0.010).

**Table 2:** Changes in BMI, waist circumference, and lipid markers after 24 weeks of treatment with pemafibrate or bezafibrate.

Variable	Pemafibrate (n = 21)		Bezafibrate (n = 21)		P value
	%Change		%Change		(%Change)
Body weight (kg)					
Baseline	81.2 ± 12.3		81.0 ± 12.1		
24 weeks	81.5 ± 12.1	0.9 ± 2.6	81.8 ± 12.4	2.4 ± 16.1	0.225
BMI (kg/m^2^)					
Baseline	28.2 ± 2.9		28.3 ± 2.8		
24 weeks	28.4 ± 2.7	0.8 ± 2.6	28.5 ± 2.9	2.3 ± 16.1	0.233
WC (cm)					
Baseline	98.4 ± 7.2		98.1 ± 7.4		
24 weeks	98.7 ± 7.1	0.4 ± 3.3	98.5 ± 7.0	2.2 ± 16.1	0.323
Triglyceride (mg/dL)					
Baseline	270.7 ± 118.7		278.6 ± 168.3		
24 weeks	152.1 ± 81.5^§^	−44.4 ± 13.8	175.8 ± 99.3^§^	−33.2 ± 24.5	0.017
HDL-C (mg/dL)					
Baseline	43.0 ± 5.4		42.9 ± 5.8		
24 weeks	51.3 ± 11.6^§^	19.1 ± 21.4	50.0 ± 12.4^§^	15.6 ± 19.2	0.458
Apo A-1 (mg/dL)					
Baseline	133.7 ± 16.5		136.2 ± 15.9		
24 weeks	144.4 ± 12.6^§^	8.9 ± 9.9	143.3 ± 15.3^§^	5.6 ± 7.8	0.115
LDL-C (mg/dL)					
Baseline	94.6 ± 22.6		89.7 ± 27.6		
24 weeks	100.9 ± 28.6	11.6 ± 36.8	102.2 ± 31.9^*^	21.0 ± 50.1	0.355
Apo B (mg/dL)					
Baseline	92.7 ± 21.1		93.4 ± 20.5		
24 weeks	83.9 ± 16.1^§^	−8.1 ± 11.8	86.7 ± 17.3^†^	−5.8 ± 6.0	0.467
RemL-C (mg/dL)					
Baseline	13.3 ± 11.0		13.5 ± 12.4		
24 weeks	5.7 ± 3.7^§^	−49.9 ± 20.1	6.6 ± 4.2^§^	−37.4 ± 28.2	0.030
Apo B-48 (μg/mL)					
Baseline	12.8 ± 11.1		10.3 ± 8.9		
24 weeks	6.4 ± 6.3^§^	−44.1 ± 29.5	5.4 ± 3.9^§^	−27.3 ± 54.9	0.010
Lp(a) (mg/dL)					
Baseline	14.8 ± 21.3		14.6 ± 22.4		
24 weeks	15.3 ± 18.2	30.2 ± 68.8	16.4 ± 20.2	34.7 ± 68.6	0.773

Variables are expressed as mean ± SD. *P < 0.05; ^†^P < 0.01; ^§^P < 0.001 vs. baseline value. BMI, body mass index; %Change, percentage change; WC, waist circumference; HDL-C, high-density lipoprotein cholesterol; Apo A-I, apolipoprotein A-I; LDL-C, low-density lipoprotein cholesterol; Apo B, apolipoprotein B; RemL-C, remnant-like particle cholesterol; Apo B-48, apolipoprotein B-48; and Lp(a), lipoprotein(a).

**Figure 2:**
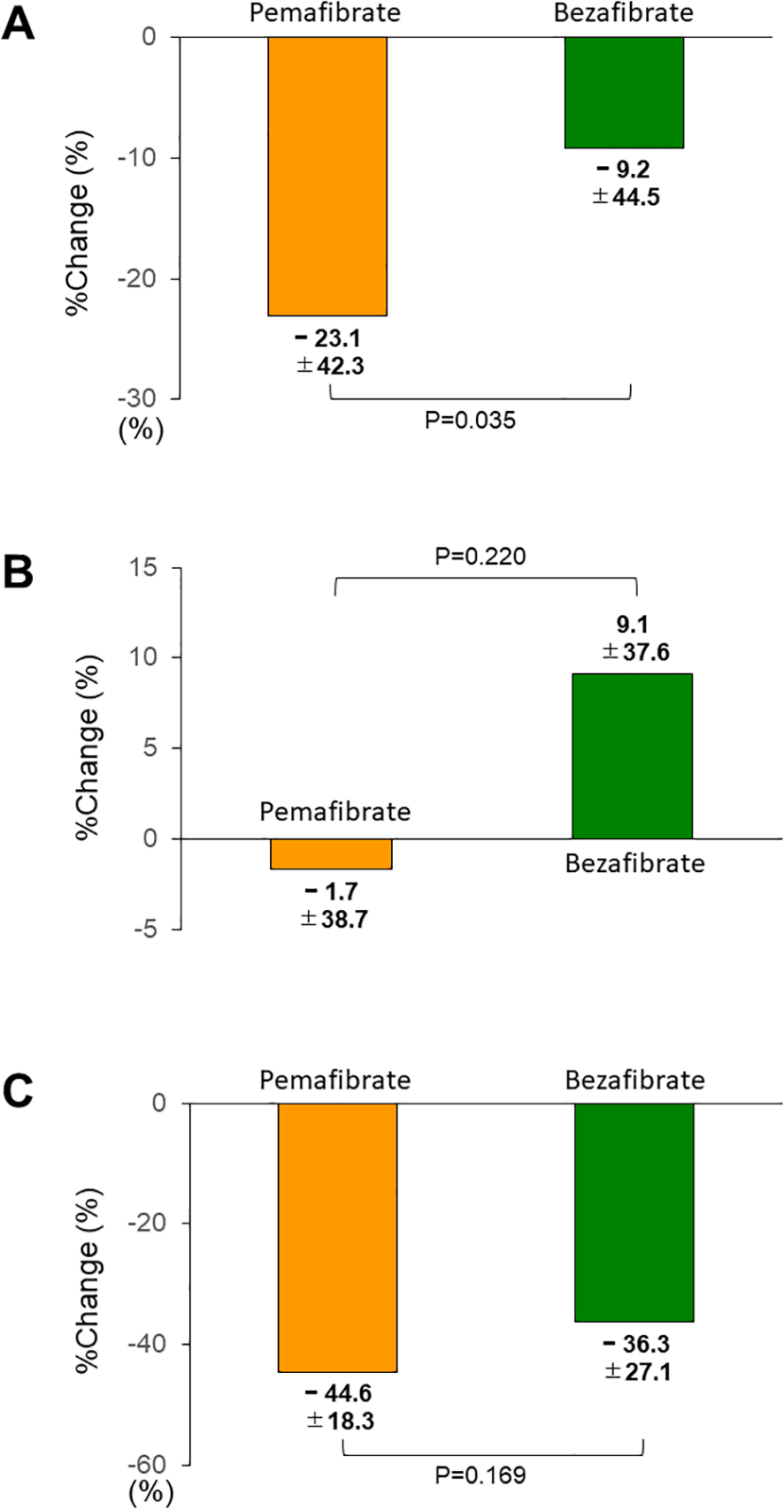
Changes in ALT **(A)**, AST **(B)**, and γGT **(C)** during pemafibrate or bezafibrate treatment. Data are expressed as mean ± SD. %Change, percentage change; ALT, alanine aminotransferase; AST, aspartate aminotransferase; and γGT, γ-glutamyltransferase.

The results of the primary outcome showed a significant decrease in serum ALT levels with pemafibrate treatment (from 36.1 ± 16.7 mg/dL to 25.2 ± 15.0 mg/dL [P < 0.001]) and no significant change before and after bezafibrate treatment (from 33.3 ± 16.9 mg/dL to 30.5 ± 23.4 mg/dL [P = 0.406]). Serum AST levels were not significantly different before and after treatment with either pemafibrate or bezafibrate (from 28.5 ± 11.9 mg/dL to 26.3 ± 12.3 mg/dL with pemafibrate [P = 0.189]; from 27.0 ± 9.8 mg/dL to 28.8 ± 13.3 mg/dL with bezafibrate [P = 0.306]). A significant decrease in serum γGT levels with both treatments was observed (from 82.3 ± 80.3 mg/dL to 38.7 ± 29.2 mg/dL with pemafibrate [P < 0.001], from 77.0 ± 62.1 mg/dL to 50.0 ± 52.9 mg/dL with bezafibrate [P < 0.001]). **Figure 2** shows the percentage change in these lipid markers during treatment with pemafibrate or bezafibrate. The percentage change in serum ALT levels was significantly greater with pemafibrate than with bezafibrate (P = 0.035) (**Figure 2**), whereas serum AST and γGT levels were not significantly different between the two treatments (AST, P = 0.220; γGT, P = 0.169) (**Figures 2B, C**).

### FLI and HSI scores before and after pemafibrate or bezafibrate treatment

**Table 3** shows the percentage changes in FLI and HSI scores before and after treatment with pemafibrate or bezafibrate. Although FLI scores significantly decreased with both treatments (both P < 0.001), the percentage change was not significantly different between the two treatments (P = 0.981). The HSI scores were not significantly different before and after treatment with either pemafibrate or bezafibrate (P = 0.739 with pemafibrate; P = 0.202 with bezafibrate).

**Table 3:** Changes in FLI and HSI scores after 24 weeks of treatment with pemafibrate or bezafibrate.

Variable	Pemafibrate (n = 21)		Bezafibrate (n = 21)		P value
	%Change		%Change		(%Change)
FLI					
Baseline	82.9 ± 13.1		77.8 ± 9.3		
24 weeks	67.2 ± 19.7^§^	−21.4 ± 13.5	61.8 ± 16.7^§^	−21.6 ± 15.4	0.983
HSI					
Baseline	37.6 ± 2.4		35.9 ± 2.9		
24 weeks	37.5 ± 3.5	0.5 ± 0.8	37.1 ± 4.7	−3.4 ± 11.0	0.211

Variables are expressed as mean ± SD. ^§^P < 0.001 vs. baseline value. FLI, fatty liver index; HSI, hepatic steatosis index; and %Change, percentage change.

### Renal function markers before and after pemafibrate or bezafibrate treatment

**Table 4** shows the percentage changes in renal function markers before and after treatment with pemafibrate or bezafibrate. Although there was a significant difference in serum creatinine levels before and after pemafibrate treatment (P = 0.006), no significant changes were observed in serum cystatin C levels, eGFRcr, or eGFRcys with pemafibrate. In contrast, significant differences in creatinine, cystatin C, eGFRcr, and eGFRcys levels were observed with bezafibrate treatment (creatinine, P < 0.001; cystatin C, P < 0.001; eGFRcr, P = 0.001; eGFRcys, P < 0.001). The percentage change in serum creatinine levels and eGFRcys was greater with bezafibrate treatment than pemafibrate treatment (creatinine, P = 0.006; eGFRcys, P = 0.030).

**Table 4:** Changes in CK, and renal function markers after 24 weeks of treatment with pemafibrate or bezafibrate.

Variable	Pemafibrate (n = 21)		Bezafibrate (n = 21)		P value
	%Change		%Change		(%Change)
CK (U/L)					
Baseline	121.4 ± 77.1		117.6 ± 56.4		
24 weeks	117.8 ± 74.1	−11.3 ± 66.5	131.1 ± 108.7	14.0 ± 83.1	0.873
Creatinine (mg/dL)					
Baseline	0.91 ± 0.22		0.89 ± 0.19		
24 weeks	0.96 ± 0.22^†^	6.5 ± 12.2	1.03 ± 0.27^§^	15.3 ± 14.8	0.006
eGFRcr (mL/min/1.73 m^2^)					
Baseline	76.0 ± 21.3		75.2 ± 18.5		
24 weeks	73.0 ± 19.5	−3.1 ± 12.5	70.4 ± 20.5^‡^	−6.6 ± 12.4	0.217
Cystatin C (mg/L)					
Baseline	1.02 ± 0.24		1.02 ± 0.28		
24 weeks	1.05 ± 0.25	3.6 ± 13.5	1.10 ± 0.28^§^	7.7 ± 11.3	0.156
eGFRcys (mL/min/1.73 m^2^)					
Baseline	75.4 ± 21.6		75.7 ± 18.2		
24 weeks	73.8 ± 19.5	−1.2 ± 13.7	70.8 ± 20.6^§^	−6.9 ± 11.2	0.030

Variables are expressed as mean ± SD. ^†^P < 0.01; ^‡^P < 0.005; ^§^P < 0.001 vs. baseline value. CK, creatine kinase; %Change, percentage change; and eGFR, estimated glomerular filtration rate.

### Glucose and insulin resistance-related markers before and after pemafibrate or bezafibrate treatment

**Table 5** shows the percentage changes in glucose and insulin resistance-related markers during pemafibrate or bezafibrate treatment. Plasma glucose levels significantly decreased with bezafibrate treatment but not with pemafibrate treatment (P = 0.018 for bezafibrate and P = 0.185 for pemafibrate). Serum insulin levels and HOMA-IR significantly decreased in both treatments (for insulin, P = 0.016 with pemafibrate and P = 0.026 with bezafibrate; for HOMA-IR, P = 0.034 with pemafibrate and P = 0.007 with bezafibrate). These decreases were more pronounced with bezafibrate treatment than with pemafibrate treatment, although the differences were not statistically significant (for insulin, P = 0.865; for HOMA-IR, P = 0.724).

**Table 5:** Changes in glucose and insulin resistance-related markers after 24 weeks of treatment with pemafibrate or bezafibrate.

Variable	Pemafibrate (n = 21)		Bezafibrate (n = 21)		P value
	%Change		%Change		(%Change)
Glucose (mg/dL)					
Baseline	115.9 ± 23.1		123.1 ± 37.9		
24 weeks	112.6 ± 21.5	−2.0 ± 12.1	115.9 ± 35.5^*^	−4.6 ± 15.1	0.407
HbA1c (%)					
Baseline	6.4 ± 0.7		6.4 ± 0.8		
24 weeks	6.2 ± 0.6	−1.5 ± 6.7	6.4 ± 0.7	−0.2 ± 5.3	0.076
Insulin (μIU/mL)					
Baseline	14.4 ± 10.3		17.0 ± 16.9		
24 weeks	11.6 ± 7.3^*^	−5.9 ± 48.9	11.8 ± 8.6^*^	−8.1 ± 58.6	0.856
HOMA-IR					
Baseline	4.4 ± 4.2		5.3 ± 5.4		
24 weeks	3.3 ± 2.3^*^	6.5 ± 52.5	3.7 ± 4.2^†^	11.2 ± 62.2	0.724

Variables are expressed as mean ± SD. *P < 0.05; ^†^P < 0.01 vs. baseline value. %Change, percentage change; HbA1c, hemoglobin A1c; and HOMA-IR, homeostasis model assessment of insulin resistance.

### FMD values before and after pemafibrate or bezafibrate treatment

**Table 6** shows the changes in FMD values, baseline and maximal brachial artery diameter, systolic and diastolic BP, and heart rate (HR) during treatment with pemafibrate or bezafibrate. FMD values, which were one of the primary outcomes, significantly increased with both treatments (P = 0.031 for pemafibrate and P < 0.001 for bezafibrate). The percentage change in the FMD values was greater with pemafibrate than with bezafibrate, although this difference was not statistically significant (P = 0.267). No significant changes were observed in systolic and diastolic BP or HR during treatment with pemafibrate or bezafibrate.

**Table 6:** Changes in FMD values, baseline and maximal diameters of the brachial artery after 24 weeks of treatment with pemafibrate or bezafibrate.

Variable	Pemafibrate (n = 21)		Bezafibrate (n = 21)		P value
	%Change		%Change		(%Change)
FMD (%)					
Baseline	5.60 ± 1.56		5.51 ± 1.76		
24 weeks	6.17 ± 21.5*	21.2 ± 22.3	6.46 ± 1.83^§^	13.4 ± 30.3	0.267
Baseline diameter (mm)					
Baseline	4.40 ± 0.56		4.20 ± 0.50		
24 weeks	4.39 ± 0.60	−0.1 ± 7.6	4.26 ± 0.50	2.9 ± 10.4	0.229
Maximal diameter (mm)					
Baseline	4.64 ± 0.57		4.43 ± 0.50		
24 weeks	4.66 ± 0.62	0.5 ± 7.9	4.54 ± 0.51	2.9 ± 10.4	0.333
Systolic BP (mmHg)					
Baseline	129 ± 27		130 ± 20		
24 weeks	124 ± 18	−0.1 ± 24.7	127 ± 22	−1.5 ± 12.1	0.748
Diastolic BP (mmHg)					
Baseline	80 ± 14		82 ± 9		
24 weeks	77 ± 14	−0.6 ± 20.4	80 ± 11	−2.2 ± 11.0	0.704
HR (beats/min)					
Baseline	63 ± 9		65 ± 14		
24 weeks	61 ± 9	−0.4 ± 18.5	67 ± 13	5.5 ± 16.3	0.206

Variables are expressed as mean ± SD. *P < 0.05; ^§^P < 0.001 vs. baseline value. %Change, percentage change; FMD, flow-mediated vasodilation; BP, blood pressure; and HR, heart rate.

## Discussion

This study examined changes in serum lipid levels, insulin resistance-related markers, and parameters related to liver damage and renal and endothelial function after 24- weeks of treatment with pemafibrate or bezafibrate in CAD patients with MASLD. The main findings were as follows. First, pemafibrate significantly reduced serum ALT levels compared to bezafibrate. The percentage changes in serum AST and γGT levels were similar between the two treatments. Second, both treatments resulted in a significant decrease in FLI, with no difference in the magnitude of change. Third, pemafibrate was comparable to bezafibrate in terms of improvement in FMD and insulin resistance-related markers. Fourth, the increase in renal function markers was significantly smaller after pemafibrate treatment than after bezafibrate treatment.

In this study, a few patients underwent non-invasive imaging, such as ultrasound or computed tomography, for fatty liver detection. Given its recommendation as a biomarker for fatty liver (1), FLI was calculated to determine the presence of hepatic steatosis in a non-invasive manner. Previous studies in Japanese and other Asian populations have shown that the FLI cut-off value for predicting MASLD is less than 60 (14, 22). On the other hand, among Japanese individuals, an FLI greater than 60 was highly sensitive (98%) and predictive (88%) for detecting MASLD (13). Using this cut-off value, patients in this study were selected to have a high probability of MASLD. In our patient population, liver biopsy was not performed to diagnose metabolic dysfunction-associated steatohepatitis (MASH). However, given the elevated FIB-4 indices, particularly in a subgroup of patients with score exceeding 2.67 and between 1.3 and 2.67, we believe that further investigation into MASH is warranted in these patients.

Patients with MASLD often have elevated serum levels of liver injury markers including ALT, AST, and γGT (23). In particular, serum ALT levels are associated with histological inflammation in MASLD, and a reduction in this marker may indicate an improvement in histological inflammation in patients with biopsy-proven MASLD (24). Since the presence of inflammation is an independent predictor of progression to advanced liver fibrosis in these patients (25), a pemafibrate-induced reduction in serum ALT levels could potentially be a therapeutic option for MASLD patients. A phase III clinical trial of pemafibrate showed a significant decrease in serum ALT levels in patients with hypertriglyceridemia treated with pemafibrate, whereas fenofibrate did not show this effect (26). Additionally, our study demonstrated that pemafibrate was more effective than bezafibrate in reducing serum ALT levels in patients with MASLD. The superiority of pemafibrate over other conventional fibrates, such as fenofibrate and bezafibrate, in improving ALT levels may be attributed to its potent and high selectivity for PPAR-α (27), which has an anti-inflammatory effect by inhibiting pro-inflammatory target genes, including nuclear factor-κB and activator protein-1 (28). Therefore, considering the decreased hepatic PPAR-α expression observed in MASLD patients (29), this selective PPAR-α modulator has emerged as a promising candidate for MASLD treatment. Activation of PPAR-α also promotes fatty acid uptake, utilization, and catabolism (30), which may contribute to the improvement of MASLD.

Insulin resistance is thought to play a critical role in the progression of MASLD (31). While the complex mechanisms underlying insulin resistance in this metabolic disorder are still not fully understood, PPARs are considered key regulators of insulin sensitivity (31). Activation of PPAR-α through fibrate medications leads to improved hepatic insulin sensitivity by increasing the expression of genes involved in glucose metabolism (30, 31). Specifically, pemafibrate has shown a potentially greater ability to reduce fasting serum glucose and insulin levels than fenofibrate, a well-established PPARα agonist known for its significant potency (26). Activation of PPAR-γ is also believed to help reduce insulin resistance by decreasing lipid storage in skeletal muscle and the liver, which is often associated with adipocyte differentiation and lipid accumulation in white adipose tissue (31). Bezafibrate, a PPAR-α agonist that also acts as a high-affinity ligand for PPAR-γ and PPAR-δ, improves insulin resistance in the liver and skeletal muscle without increasing insulin secretion in patients with type 2 DM and dyslipidemia (32). Despite the potential advantages of activating both PPAR-α and γ (33), a comparative study of the therapeutic effects of a highly selective PPAR-α agonist versus a pan-PPAR agonist on insulin resistance in MASLD patients is essential to assess their effectiveness in this specific patient group. Our retrospective study suggests that pemafibrate may be as effective as bezafibrate in improving insulin resistance in patients with MASLD, as assessed by HOMA-IR and serum insulin levels. Prospective studies directly comparing pemafibrate with other fibrates are needed to confirm these findings and to determine the optimal therapeutic approach for this population.

Endothelial dysfunction significantly contributes to the development of atherosclerosis and plays a crucial role in its onset, progression, and continuation, ultimately leading to cardiovascular events, including CAD and stroke (4). Several studies using brachial FMD to evaluate vascular endothelial function have shown impaired endothelial function in patients with MASLD (4, 10) which can be improved by fibrate treatment (34). Previous research has indicated that the enhancement of FMD is due to various mechanisms notably, the upregulation of endothelial nitric oxide (eNOS) and the production of nitric oxide (NO), which play significant roles (35). However, the mechanisms by which activation of PPAR-α and PPAR-γ regulate endothelial NO production through eNOS activity and expression remain incompletely understood. While PPAR-α is known to increase eNOS protein expression by stabilizing its mRNA (36), PPAR-γ appears to enhance eNOS activity through mechanisms that do not involve transcription, without altering eNOS mRNA levels (37). Despite these differences, our retrospective analysis found no significant difference in the impact of pemafibrate (a highly selective PPAR-α agonist) and bezafibrate (a PPAR-α/γ dual agonist) on endothelial function in patients with MASLD and CAD. Further research is necessary to clarify the intricate relationship between PPARs and the eNOS/NO pathway, and to develop more effective PPAR-based treatments for these patients. Pemafibrate treatment improved vascular endothelial dysfunction in diabetic mice by reducing vasoconstrictor eicosanoids (38). However, its benefits are not clear when used alone in hypertensive and insulin-resistant rats (39). Currently, data on the effects of pemafibrate on endothelial function are scarce and no clinical trials have been conducted to date. To our knowledge, this study is the first to demonstrate the efficacy of pemafibrate treatment in enhancing endothelial function, as assessed by FMD measurements in humans. Our findings suggest that even in patients with established atherosclerosis and ongoing medical therapy, selective PPAR-α modulation may provide further stabilization of endothelial function. This implies that fibrates could serve as a valuable add-on therapy in a ‘real-world’ clinical setting for complex patients with multi-metabolic disorders.

Several studies have shown a strong association between MASLD and increased risk of developing chronic kidney disease (CKD) (40). While studies have linked MASLD and CKD, the underlying mechanisms remain unclear (41). Compared to other fibrates such as bezafibrate and fenofibrate, pemafibrate is primarily excreted via the bile, potentially reducing the risk of renal impairment. Our findings support this hypothesis, as patients treated with pemafibrate experienced smaller increases in serum creatinine and slower declines in eGFRcys compared to those treated with bezafibrate. Although this study did not assess proteinuria, a previous study suggested that pemafibrate may reduce proteinuria in patients with CKD (41). Further studies are warranted to explore the potential renal protective effects of pemafibrate in this patient population.

Although it is controversial whether MASLD is an independent risk factor for cardiovascular events, many studies have consistently reported an increased risk of cardiovascular events in patients with MASLD (4). Therefore, effective management of MASLD through lifestyle modifications or treatment strategies could potentially prevent the progression of these events, including CAD. One promising treatment approach involves fibrate medications such as pemafibrate, which has been shown to improve liver fibrosis in patients with MASLD, as assessed by magnetic resonance elastography (42). In the PROMINENT trial, pemafibrate treatment was not found to be effective in preventing cardiovascular events in diabetic patients with hypertriglyceridemia; however, it significantly reduced the risk of developing MASLD (43). In this study, the benefit of improving MASLD in the prognosis of patients with CAD remains unclear. This finding highlights the need for further research on interventions targeting both MASLD and CAD.

This study has several limitations. First, it was a *post-hoc* analysis conducted at a single center with a small sample size, indicating that further studies with larger patient populations are necessary to validate our findings.

This study has several limitations. First, it was a *post-hoc* analysis conducted at a single center with a small sample size. Due to this limited sample size, it should be noted that the significant differences observed in the pooled data for ALT and creatinine were not consistently replicated when analyzing Period 1 and Period 2 separately. For instance, the difference in ALT was primarily evident in Period 2, and the difference in creatinine did not reach statistical significance in either individual period (**Supplementary Table 3**). These discrepancies are likely attributable to a loss of statistical power in the period-specific analyses. While the crossover design was intended to minimize inter-individual variability, these findings should be interpreted with caution, indicating that further studies with larger patient populations are necessary to validate our findings. Second, this study was constrained by the absence of a definitive diagnosis of MASLD based on established criteria, which necessitated imaging or histological evidence of hepatic steatosis in the absence of secondary causes of hepatic fat accumulation. To mitigate this limitation, our study used MASLD indices, such as FLI and HSI, which have been used in previous studies as surrogate markers for MASLD (11, 12). Third, the study’s findings may not be generalizable to more diverse populations because of the limited racial and ethnic diversity in the study sample. In addition, the participants in this study were almost exclusively male (20 out of 21). Previous research has highlighted significant sexual dimorphism in MASLD, where premenopausal women often show a lower prevalence of advanced fibrosis compared to men, likely due to the protective effects of estrogen on hepatic stellate cells and lipid metabolism (44). In our study, the nearly exclusive inclusion of male participants limits the applicability of the findings to women, particularly considering that the activation of PPAR-α and its subsequent effect on endothelial function may be modulated by sex hormones.

In conclusion, pemafibrate outperformed bezafibrate in reducing serum ALT levels without adversely affecting renal function in CAD patients with MASLD. Additionally, it was as effective as bezafibrate in improving insulin resistance and endothelial dysfunction. These findings suggest that pemafibrate could offer a superior treatment approach compared to bezafibrate for individuals with metabolic disorders, such as MASLD.

## Data Availability

The original contributions presented in the study are included in the article/**Supplementary Material**. Further inquiries can be directed to the corresponding author.

## Supplementary material

Supplementary Table 1 Changes in BMI, waist circumference, and lipid markers after pemafibrate or bezafibrate treatment during Period 1 and Period 2.

Supplementary Table 2 Changes in FLI and HIS after pemafibrate or bezafibrate treatment during Period 1 and Period 2.

Supplementary Table 3 Changes in CK, liver enzymes, and renal function after pemafibrate or bezafibrate treatment during Period 1 and Period 2.

Supplementary Table 4 Changes in glucose and insulin resistance-related markers after pemafibrate or bezafibrate treatment during Period 1 and Period 2.

Supplementary Table 5 Changes in FMD values, baseline and maximal diameters of the brachial artery after pemafibrate or bezafibrate treatment during Period 1 and Period 2.

## References

[1] Eslam M Newsome PN Sarin SK Anstee QM Targher G Romero-Gomez M . A new definition for metabolic dysfunction-associated fatty liver disease: an international expert consensus statement. J Hepatol. (2020) 73:202–9. doi: 10.1016/j.jhep.2020.03.039, PMID: 32278004

[2] Powell EE Wong VW Rinella M . Non-alcoholic fatty liver disease. Lancet. (2021) 397:2212–24. doi: 10.1016/S0140-6736(20)32511-3, PMID: 33894145

[3] Byrne CD Targer G . Non-alcoholic fatty liver disease-related risk of cardiovascular disease and other cardiac complications. Diabetes Obes Metab. (2022) 24:28–43. doi: 10.1111/dom.14484, PMID: 34324263

[4] Ozturk K Uygun A Guler AK Demirci H Ozdemir C Cakir M . Nonalcoholic fatty liver disease is an independent risk factor for atherosclerosis in young adult man. Atherosclerosis. (2015) 240:380–6. doi: 10.1016/j.atherosclerosis.2015.04.009, PMID: 25875390

[5] Farrell GC Larter CZ . Nonalcoholic fatty liver disease: from steatosis to cirrhosis. Hepatology. (2006) 43:S99–112. doi: 10.1002/hep.20973, PMID: 16447287

[6] Morishita A Oura K Takuma K Nakahara M Tadokoro T Fujita K . Pemafibrate improves liver dysfunction and non-invasive surrogates for liver fibrosis in patients with non-alcoholic fatty liver disease with hypertriglyceridemia: a multicenter study. Hepatol Int. (2023) 17:606–14. doi: 10.1007/s12072-022-10453-1, PMID: 36583842 PMC10224826

[7] Teramoto T Shirai K Daida H Yamada N . Effects of bezafibrate on lipid and glucose metabolism in dyslipidemic patients with diabetes: the J-BENEFIT study. Cardiovasc Diabetol. (2012) 11:29. doi: 10.1186/1475-2840-11-29, PMID: 22439599 PMC3342914

[8] Bezafibrate Infarction Prevention (BIP) Study Group . Secondary prevention by raising HDL cholesterol and reducing triglycerides in patients with coronary artery disease. Circulation. (2000) 102:21–7. doi: 10.1161/01.cir.102.1.21, PMID: 10880410

[9] Nakamura A Kagaya Y Saito H Kanazawa M Sato K Miura M . Efficacy and safety of pemafibrate versus bezafibrate to treat patients with hypertriglyceridemia: a randomized crossover study. J Atheroscler Thromb. (2023) 30:443–54. doi: 10.5551/jat.63659, PMID: 35768226 PMC10164592

[10] Colak Y Senates E Yesil A Yilmaz Y Ozturk O Doganay L . Assessment of endothelial function in patients with nonalcoholic fatty liver disease. Endocrine. (2013) 43:100–7. doi: 10.1007/s12020-012-9712-1, PMID: 22661277

[11] Bourgonje AR van den Berg EH Kieneker LM Nilsen T Hidden C Bakker SJL . Plasma calprotectin levels associate with suspected metabolic-associated fatty liver disease and all-cause mortality in the general population. Int J Mol Sci. (2022) 23:15708. doi: 10.3390/ijms232415708, PMID: 36555350 PMC9778771

[12] van den Berg EH Corsetti JP Bakker SJL Dullaart RPF . Plasma apoE elevations are associated with NAFLD: the PREVEND study. PloS One. (2019) 14:e0220659. doi: 10.1371/journal.pone.0220659, PMID: 31386691 PMC6684074

[13] Murayama K Okada M Tanaka K Inadomi C Yoshioka W Kubotsu Y . Prediction of nonalcoholic fatty liver disease using noninvasive and non-imaging procedures in Japanese health checkup examinees. Diagnostics (Basel). (2021) 11:132. doi: 10.3390/diagnostics11010132, PMID: 33467114 PMC7830542

[14] Takahashi S Tanaka M Higashiura Y Mori K Hanawa N Ohnishi H . Prediction and validation of nonalcoholic fatty liver disease by fatty liver index in a Japanese population. Endocr J. (2022) 69:463–71. doi: 10.1507/endocrj.EJ21-0563, PMID: 34803123

[15] Bedogni G Bellentani S Miglioli L Masutti F Passalacqua M Castiglione A . The fatty liver index: a simple and accurate predictor of hepatic steatosis in the general population. BMC Gastroenterol. (2006) 6:33. doi: 10.1186/1471-230X-6-33, PMID: 17081293 PMC1636651

[16] Lee JH Kim D Kim HJ Lee CH Yang JI Kim W . Hepatic steatosis index: a simple screening tool reflecting nonalcoholic fatty liver disease. Dig Liver Dis. (2010) 42:503–8. doi: 10.1016/j.dld.2009.08.002, PMID: 19766548

[17] Sterling RK Lissen E Clumeck N Sola R Correa MC Montaner J . Development of a simple noninvasive index to predict significant fibrosis in patients with HIV/HCV coinfection. Hepatology. (2006) 43:1317–25. doi: 10.1002/hep.21178, PMID: 16729309

[18] American Diabetes Association . Standards of medical care in diabetes–2010. Diabetes Care. (2010) 33:S11–61. doi: 10.2337/dc10-S011, PMID: 20042772 PMC2797382

[19] Matsuo S Imai E Horio M Yasuda Y Tomita K Nitta K . Collaborators developing the Japanese equation GFR: Revised equations for estimated GFR from serum creatinine in Japan. Am J Kidney Dis. (2009) 53:982–92. doi: 10.1053/j.ajkd.2008.12.034, PMID: 19339088

[20] Horio M Imai E Yasuda Y Watanabe T Matsuo S . GFR estimation using standardized serum cystatin C in Japan. Am J Kidney Dis. (2013) 61:197–203. doi: 10.1053/j.ajkd.2012.07.007, PMID: 22892396

[21] Corretti MC Anderson TJ Benjamin EJ Celermajer D Charbonneau F Creager MA . Guidelines for the ultrasound assessment of endothelial-dependent flow-mediated vasodilation of the brachial artery: a report of the International Brachial Artery Reactivity Task Force. J Am Coll Cardiol. (2002) 39:257–65. doi: 10.1016/s0735-1097(01)01746-6, PMID: 11788217

[22] Huang X Xu M Chen Y Peng K Huang Y Wang P . Validation of the fatty liver index for nonalcoholic fatty liver disease in middle-aged and elderly Chinese. Med (Baltimore). (2015) 94:e1682. doi: 10.1097/MD.0000000000001682, PMID: 26448014 PMC4616754

[23] Hanley AJG Williams K Festa A Wagenknecht LE D’Agostino RB Jr Kempf J . Elevations in markers of liver injury and risk of type 2 diabetes: The insulin resistance atherosclerosis study. Diabetes. (2004) 53:2623–32. doi: 10.2337/diabetes.53.10.2623, PMID: 15448093

[24] Seko Y Sumida Y Tanaka S Mori K Taketani H Ishiba H . Serum alanine aminotransferase predicts the histological course of non-alcoholic steatohepatitis in Japanese patients. Hepatol Res. (2015) 45:E53–61. doi: 10.1111/hepr.12456, PMID: 25429984

[25] Argo CK Northup PG AI-Osaimi AMS Caldwell SH . Systematic review of risk factors for fibrosis progression in non-alcoholic steatohepatitis. J Hepatol. (2009) 51:371–9. doi: 10.1016/j.jhep.2009.03.019, PMID: 19501928

[26] Arai H Yamashita S Yokote K Araki E Suganami H Ishibashi S . Efficacy and safety of pemafibrate versus fenofibrate in patients with high triglyceride and low HDL cholesterol levels: a multicenter, placebo-controlled, double-blind, randomized trial. J Atheroscler Thromb. (2018) 25:521–38. doi: 10.5551/jat.44412, PMID: 29628483 PMC6005227

[27] Yamashita S Masuda D Matsuzawa Y . Pemafibrate, a new selective PPARα modulator: drug concept and its clinical applications for dyslipidemia and metabolic diseases. Curr Atheroscler Rep. (2020) 22:5. doi: 10.1007/s11883-020-0823-5, PMID: 31974794 PMC6978439

[28] Xu X Otsuki M Saito H Sumitani S Yamamoto H Asanuma N . PPARalpha and GRR differentially down-regulate the expression of nuclear factor-kappaB-responsive genes in vascular endothelial cells. Endocrinology. (2001) 142:3332–9. doi: 10.1210/endo.142.8.8340, PMID: 11459775

[29] Fujita K Nozaki Y Wada K Yoneda M Fujimoto Y Fujitake M . Dysfunction very-low-density lipoprotein synthesis and release is a key factor in nonalcoholic steatohepatitis pathogenesis. Hepatology. (2009) 50:772–80. doi: 10.1002/hep.23094, PMID: 19650159

[30] Kersten S . Integrated physiology and systems biology of PPARalpha. Mol Metab. (2014) 3:354–71. doi: 10.1016/j.molmet.2014.02.002, PMID: 24944896 PMC4060217

[31] Ferré P . The biology of peroxisome proliferator-activated receptors: relationship with lipid metabolism and insulin sensitivity. Diabetes. (2004) 53:S43–50. doi: 10.2337/diabetes.53.2007.s43, PMID: 14749265

[32] Shiochi H Ohkura T Fujioka Y Sumi K Yamamoto N Nakanishi R . Bezafibrate improves insulin resistance evaluated using the glucose clamp technique in patients with type 2 diabetes mellitus: a small-scale clinical study. Diabetol Metab Syndr. (2014) 6:113. doi: 10.1186/1758-5996-6-113, PMID: 25360162 PMC4213459

[33] Fernandes-Santos C Carneiro RE de Souza Mendonca L Aguila MB Mandarim-de-Lacerda CA . Pan-PPAR agonist beneficial effects in overweight mice fed a high-fat high-sucrose diet. Nutrition. (2009) 25:818–27. doi: 10.1016/j.nut.2008.12.010, PMID: 19268533

[34] Sahebkar A Giua R Pedone C Ray KK Vallejo-Vaz AJ Costanzo L . Fibrate therapy and flow-mediated dilation: a systematic review and meta-analysis of randomized placebo-controlled trials. Pharmacol Res. (2016) 111:163–79. doi: 10.1016/j.phrs.2016.06.011, PMID: 27320045

[35] Green DJ Dawson EA Groenewoud HMM Jones H Thijssen DHJ . Is flow-mediated dilation nitric oxide mediated? A meta-analysis. Hypertension. (2014) 63:376–82. doi: 10.1161/HYPERTENSIONAHA.113.02044, PMID: 24277765

[36] Goya K Sumitani S Xu X Kitamura T Yamamoto H Kurebayashi S . Peroxisome proliferator-activated receptor α agonists increase nitric oxide synthase expression in vascular endothelial cells. Arterioscler Thromb Vasc Biol. (2004) 24:658–63. doi: 10.1161/01.ATV.0000118682.58708.78, PMID: 14751809

[37] Calnek DS Mazzella L Roser S Roman J Hart CM . Peroxisome proliferator-activated receptor gamma ligands increase release of nitric oxide from endothelial cells. Arterioscler Thromb Vasc Biol. (2003) 23:52–7. doi: 10.1161/01.atv.0000044461.01844.c9, PMID: 12524224

[38] Suto K Fukuda D Shinohara M Ganbaatar B Yagi S Kusunose K . Pemafibrate, a novel selective peroxisome proliferator-activated receptor α modulator, reduces plasma eicosanoid levels and ameliorates endothelial dysfunction in diabetic mice. J Atheroscler Thromb. (2021) 28:1349–60. doi: 10.5551/jat.61101, PMID: 33775978 PMC8629704

[39] Yoshida M Nakamura K Miyoshi T Yoshida M Kondo M Akazawa K . Combination therapy with pemafibrate (K-877) and pitavastatin improves vascular endothelial dysfunction in dahl/salt-sensitive rats fed a high-salt and high-fat diet. Cardiovasc Diabetol. (2020) 19:149. doi: 10.1186/s12933-020-01132-2, PMID: 32979918 PMC7520032

[40] Bilson J Mantovani A Byrne CD Targher G . Steatotic liver disease, MASLD and risk of chronic kidney disease. Diabetes Metab. (2024) 50:101506. doi: 10.1016/j.diabet.2023.101506, PMID: 38141808

[41] Seki M Nakano T Tanaka S Matsukuma Y Funakoshi K Ohkuma T . Design and methods of an open-label, randomized controlled trial to evaluate the effect of pemafibrate on proteinuria in CKD patients (PROFIT-CKD). Clin Exp Nephrol. (2023) 27:358–64. doi: 10.1007/s10157-023-02322-4, PMID: 36738362

[42] Nakajima A Eguchi Y Yoneda M Imajo K Tamaki N Suganami H . Randomised clinical trial: Pemafibrate, a novel selective peroxisome proliferator-activated receptor α modulator (SPPARMα), versus placebo in patients with non-alcoholic fatty liver disease. Aliment Pharmacol Ther. (2021) 54:1263–77. doi: 10.1111/apt.16596, PMID: 34528723 PMC9292296

[43] Pradhan AD Glynn RJ Fruchart JC MacFadyen JG Zaharris ES Everett BM . Triglyceride lowering with pemafibrate to reduce cardiac risk. N Engl J Med. (2022) 387:1923–34. doi: 10.1056/NEJMoa2210645, PMID: 36342113

[44] Cherubini A Della TS Pelusi S Valenti L . Sexual dimorphism of metabolic dysfunction-associated steatotic liver disease. Trends Mol Med. (2024) 30:1126–36. doi: 10.1016/j.molmed.2024.05.013, PMID: 38890029

