# Supplementary material for: Comparative effectiveness of pemafibrate versus bezafibrate on hepatic and vascular endothelial function in patients with coronary artery disease and metabolic dysfunction-associated steatotic liver disease
Source: Front Endocrinol (Lausanne). 2026 Feb 11;17:1750308. doi: 10.3389/fendo.2026.1750308 (PMC12932250; doi:10.3389/fendo.2026.1750308)
Supplement: Supplementary Table 1 — Changes in BMI, waist circumference, and lipid markers after pemafibrate or bezafibrate treatment during Period 1 and Period 2. [file Table1.docx]

Supplemental TABLE 1 Changes in BMI, waist circumference, and lipid markers after pemafibrate or bezafibrate treatment during Period 1 and Period 2.

| Variable | Period 1 | |  | Period 2 | | P value (%Change) |
| --- | --- | --- | --- | --- | --- | --- |
|  | Pemafibrate  (n = 9) | Bezafibrate (n = 12) | P value (%Change) | Pemafibrate  (n = 12) | Bezafibrate (n = 9) |  |
| BMI (kg/m^2^) |  |  |  |  |  |  |
| Baseline | 28.6 ± 2.8 | 27.7 ± 2.1 |  | 28.0 ± 2.6 | 29.1 ± 3.7 |  |
| 24 weeks | 28.4 ± 2.7 | 27.8 ± 2.0 |  | 27.6 ± 2.4 | 29.6 ± 3.3 |  |
| %Change | −0.7 ± 1.7 | 0.1 ± 2.7 | 0.269 | −0.5 ± 1.5 | 1.7 ± 2.5 | 0.159 |
| WC (cm) |  |  |  |  |  |  |
| Baseline | 100.0 ± 6.6 | 96.0 ± 5.8 |  | 97.6 ± 6.8 | 100.1 ± 10.0 |  |
| 24 weeks | 98.9 ± 6.0 | 97.2 ± 4.5 |  | 95.9 ± 5.4 | 100.8 ± 9.6 |  |
| %Change | −1.1 ± 2.8 | 1.5 ± 2.7 | 0.269 | −0.6 ± 1.5 | 1.7 ± 2.6 | 0.087 |
| Triglyceride (mg/dL) |  |  |  |  |  |  |
| Baseline | 277.9 ± 123.0 | 227.0 ± 106.5 |  | 236.4 ± 70.4 | 329.7 ± 201.7 |  |
| 24 weeks | 168.7 ± 91.1^†^ | 162.3 ± 77.6^⁎^ |  | 127.9 ± 44.8^†^ | 182.2 ± 86.4^†^ |  |
| %Change | −40.1 ± 15.4 | −27.5 ± 18.7 | 0.261 | −45.9 ± 9.7 | −38.4 ± 29.3 | 0.905 |
| HDL-C (mg/dL) |  |  |  |  |  |  |
| Baseline | 43.3 ± 5.7 | 41.5 ± 5.4 |  | 43.4 ± 5.4 | 44.1 ± 5.2 |  |
| 24 weeks | 54.7 ± 13.7 | 46.6 ± 10.2^⁎^ |  | 53.5 ± 12.9^⁎^ | 50.0 ± 7.9^†^ |  |
| %Change | 25.0 ± 21.1 | 12.4 ± 22.0 | 0.160 | 22.2± 19.3 | 13.3 ± 11.3 | 0.413 |
| Apo A-1 (mg/dL) |  |  |  |  |  |  |
| Baseline | 128.4 ± 16.1 | 129.4 ± 10.3 |  | 143.3 ± 15.5 | 137.2 ± 16.3 |  |
| 24 weeks | 141.5 ± 15.1^⁎^ | 140.8 ± 8.8^⁎^ |  | 147.3 ± 8.7 | 142.9 ± 14.4^†^ |  |
| %Change | 10.9 ± 11.0 | 9.1 ± 6.3 | 0.331 | 3.6 ± 8.6 | 4.4 ± 3.0 | 0.182 |
| LDL-C (mg/dL) |  |  |  |  |  |  |
| Baseline | 90.8 ± 22.8 | 96.3 ± 16.2 |  | 95.3 ± 44.0 | 92.8 ± 13.9 |  |
| 24 weeks | 93.3 ± 15.8 | 104.2 ± 18.4 |  | 113.6 ± 33.3 | 98.3 ± 28.6 |  |
| %Change | 3.6± 50.0 | 11.7 ± 29.8 | 0.261 | 10.6 ± 44.6 | 29.1± 36.2 | 0.447 |
| Apo B (mg/dL) |  |  |  |  |  |  |
| Baseline | 91.5 ± 21.8 | 86.2 ± 11.1 |  | 88.4 ± 13.2 | 94.8 ± 31.4 |  |
| 24 weeks | 81.3 ± 13.8^⁎^ | 86.9 ± 13.9 |  | 85.1 ± 7.7 | 93.3 ± 21.3 |  |
| %Change | −9.4 ± 9.2 | 2.1 ± 19.2 | 0.295 | −1.5 ± 18.3 | −1.8 ± 15.5 | 0.450 |
| RemL-C (mg/dL) |  |  |  |  |  |  |
| Baseline | 12.3 ± 7.4 | 16.3 ± 18.4 |  | 9.8 ± 2.1 | 17.1 ± 13.7 |  |
| 24 weeks | 6.9 ± 4.6^†^ | 5.6 ± 2.5^⁎^ |  | 4.5 ± 1.7^†^ | 7.8 ± 5.1 |  |
| %Change | −41.3 ± 20.8 | −38.4 ± 35.4 | 0.046 | −53.4 ± 12.5 | −40.1 ± 31.2 | 0.447 |
| Apo B-48 (μg/mL) |  |  |  |  |  |  |
| Baseline | 9.8 ± 8.0 | 11.6 ± 8.9 |  | 11.8 ± 11.1 | 8.5 ± 5.8 |  |
| 24 weeks | 8.2 ± 9.6 | 5.1 ± 2.0^⁎^ |  | 4.6 ± 3.5^†^ | 4.9 ± 3.2 |  |
| %Change | −31.8 ± 26.4 | −33.3 ± 60.2 | 0.230 | −55.6 ± 18.1 | −22.5 ± 43.7 | 0.156 |
| Lp(a) (mg/dL) |  |  |  |  |  |  |
| Baseline | 15.7 ± 18.6 | 17.4 ± 5.8 |  | 15.4 ± 26.1 | 16.4 ± 19.9 |  |
| 24 weeks | 17.0 ± 16.3 | 19.6 ± 7.2^⁎^ |  | 16.8 ± 21.6 | 20.4 ± 25.0 |  |
| %Change | 13.6 ± 47.0 | 16.3 ± 38.4 | 0.180 | 18.7 ± 35.6 | 23.3 ± 45.1 | 0.090 |

Variables are expressed as mean ± SD. ^⁎^P < 0.05; ^†^P < 0.01 vs. baseline value.

Period 1: Treatment period prior to washout; Period 2: Treatment period subsequent to washout. BMI, body mass index; %Change, percentage change; WC, waist circumference; HDL-C, high-density lipoprotein cholesterol; Apo A-I, apolipoprotein A-I; LDL-C, low-density lipoprotein cholesterol; Apo B, apolipoprotein B; RemL-C, remnant-like particle cholesterol; Apo B-48, apolipoprotein B-48; and Lp(a), lipoprotein(a).

Supplemental TABLE 2 Changes in FLI and HIS after pemafibrate or bezafibrate treatment during Period 1 and Period 2.

| Variable | Period 1 | |  | Period 2 | | P value (%Change) |
| --- | --- | --- | --- | --- | --- | --- |
|  | Pemafibrate  (n = 9) | Bezafibrate (n = 12) | P value (%Change) | Pemafibrate  (n = 12) | Bezafibrate (n = 9) |  |
| FLI |  |  |  |  |  |  |
| Baseline | 83.9 ± 10.3 | 76.2 ± 9.9 |  | 81.6 ± 15.2 | 79.3 ± 8.5 |  |
| 24 weeks | 66.1 ± 16.4^†^ | 62.2 ± 16.4^†^ |  | 67.3 ± 24.0^⁎^ | 61.4 ± 17.0^†^ |  |
| %Change | −22.4 ± 11.7 | −19.6 ± 13.2 | 0.551 | −31.0 ± 29.1 | −23.4 ± 16.2 | 0.968 |
| HSI |  |  |  |  |  |  |
| Baseline | 37.5 ± 2.1 | 36.3 ± 2.0 |  | 37.7 ± 2.8 | 35.5 ± 3.5 |  |
| 24 weeks | 37.0 ± 2.9 | 38.1 ± 4.3 |  | 38.1 ± 3.8 | 36.2 ± 4.7 |  |
| %Change | −1.2 ± 7.1 | 5.2 ± 11.7 | 0.331 | 0.4 ± 8.1 | 1.8 ± 9.5 | 0.549 |

Variables are expressed as mean ± SD. ^⁎^P < 0.05; ^†^P < 0.01 vs. baseline value.

Period 1: Treatment period prior to washout; Period 2: Treatment period subsequent to washout. FLI, fatty liver index; HSI, hepatic steatosis index; and %Change, percentage change.

Supplemental TABLE 3 Changes in CK, liver enzymes, and renal function after pemafibrate or bezafibrate treatment during Period 1 and Period 2.

| Variable | Period 1 | |  | Period 2 | | P value (%Change) |
| --- | --- | --- | --- | --- | --- | --- |
|  | Pemafibrate  (n = 9) | Bezafibrate (n = 12) | P value (%Change) | Pemafibrate  (n = 12) | Bezafibrate (n = 9) |  |
| CK (U/L) |  |  |  |  |  |  |
| Baseline | 104.6 ± 45.1 | 156.1 ± 122.0 |  | 123.7 ± 60.5 | 106.7 ± 51.3 |  |
| 24 weeks | 100.7 ± 37.1 | 123.6 ± 51.3 |  | 102.8 ± 55.1 | 144.9 ± 116.3 |  |
| %Change | −1.52 ± 27.1 | −12.6 ± 70.3 | 0.968 | −9.1 ± 36.4 | 39.1 ± 97.7 | 0.243 |
| AST (U/L) |  |  |  |  |  |  |
| Baseline | 34.8 ± 10.7 | 28.8 ± 6.1 |  | 20.1 ± 5.2 | 25.0 ± 14.3 |  |
| 24 weeks | 31.9 ± 14.1 | 22.8 ± 8.1 |  | 24.6 ± 8.6 | 30.0 ± 16.6 |  |
| %Change | −14.2 ± 36.0 | −8.2 ± 29.2 | 0.589 | 7.6 ± 46.7 | 13.7 ± 14.7 | 0.185 |
| ALT (U/L) |  |  |  |  |  |  |
| Baseline | 42.6 ± 16.7 | 40.9 ± 10.3 |  | 27.4 ± 10.2 | 23.3 ± 9.4 |  |
| 24 weeks | 28.7 ± 17.1^†^ | 25.3 ± 16.0 |  | 21.9 ± 10.0^⁎^ | 24.1 ± 14.4 |  |
| %Change | −29.4 ± 35.5 | −28.5 ± 32.5 | 0.370 | −20.7 ± 21.2 | 7.3 ± 54.3 | 0.029 |
| γ-GT (U/L) |  |  |  |  |  |  |
| Baseline | 80.5 ± 38.2 | 65.0 ± 26.1 |  | 41.7 ± 39.5 | 45.6 ± 26.6 |  |
| 24 weeks | 39.5 ± 14.3^†^ | 34.2 ± 16.3^†^ |  | 36.7 ± 29.7^†^ | 25.8 ± 13.1^†^ |  |
| %Change | −46.0 ± 16.7 | −45.6 ± 18.0 | 0.968 | −39.8 ± 15.0 | −37.4 ± 20.6 | 0.219 |
| Creatinine (mg/dL) |  |  |  |  |  |  |
| Baseline | 0.96 ± 0.22 | 0.86 ± 0.18 |  | 0.85 ± 0.20 | 0.93 ± 0.24 |  |
| 24 weeks | 0.99 ± 0.22 | 0.88 ± 0.22 |  | 0.98 ± 0.23^⁎^ | 0.97 ± 0.21 |  |
| %Change | 3.8 ± 11.21 | 2.4 ± 7.5 | 0.824 | 15.6 ± 16.1 | 4.8 ± 7.9 | 0.133 |
| eGFRcr (mL/min/1.73m^2^) |  |  |  |  |  |  |
| Baseline | 66.0 ± 15.2 | 81.0 ± 18.6 |  | 83.9 ± 22.6 | 75.4 ± 23.6 |  |
| 24 weeks | 65.9 ± 13.1 | 77.1 ± 20.4 |  | 76.8 ± 21.4 | 73.5 ± 20.8 |  |
| %Change | −0.8 ± 10.4 | −5.3 ± 11.0 | 0.412 | −7.4 ± 16.0 | −1.3 ± 10.7 | 0.549 |
| Cystatin C (mg/L) |  |  |  |  |  |  |
| Baseline | 1.11 ± 0.20 | 0.96 ± 0.19 |  | 0.95 ± 0.20 | 1.06 ± 0.29 |  |
| 24 weeks | 1.10 ± 0.19 | 1.02 ± 0.27 |  | 1.01 ± 0.25 | 1.07 ± 0.29 |  |
| %Change | −0.2 ± 7.7 | 5.9 ± 10.9 | 0.206 | 7.7 ± 20.6 | 2.0 ± 10.8 | 0.780 |
| eGFRcys (mL/min/1.73m^2^) |  |  |  |  |  |  |
| Baseline | 65.3 ± 14.9 | 79.0 ± 20.0 |  | 84.1 ± 22.7 | 75.4 ± 23.6 |  |
| 24 weeks | 66.2 ± 13.0 | 77.2 ± 20.4 |  | 80.5 ± 20.7 | 73.8 ± 20.9 |  |
| %Change | 2.2 ± 10.5 | −1.7 ± 16.2 | 0.272 | −5.6 ± 16.4 | −1.0 ± 10.7 | 0.761 |

Variables are expressed as mean ± SD. ^⁎^P < 0.05; ^†^P < 0.01 vs. baseline value.

Period 1: Treatment period prior to washout; Period 2: Treatment period subsequent to washout. CK, creatine kinase; %Change, percentage change; and eGFR, estimated glomerular filtration rate.

Supplemental TABLE 4 Changes in glucose and insulin resistance-related markers after pemafibrate or bezafibrate treatment during Period 1 and Period 2.

| Variable | Period 1 | |  | Period 2 | | P value (%Change) |
| --- | --- | --- | --- | --- | --- | --- |
|  | Pemafibrate  (n = 9) | Bezafibrate (n = 12) | P value (%Change) | Pemafibrate  (n = 12) | Bezafibrate (n = 9) |  |
| Glucose  (mg/dL) |  |  |  |  |  |  |
| Baseline | 119.0 ± 32.3 | 115.9 ± 19.6 |  | 119.2 ± 16.5 | 109.5 ± 14.3 |  |
| 24 weeks | 110.2 ± 17.5 | 119.8 ± 26.4 |  | 118.2 ± 21.2 | 103.8 ± 16.5 |  |
| %Change | −3.7 ± 16.5 | 3.0 ± 10.5 | 0.766 | −1.0 ± 10.4 | −5.4 ± 6.3 | 0.175 |
| HbA1c (%) |  |  |  |  |  |  |
| Baseline | 6.4 ± 0.8 | 6.6 ± 0.9 |  | 6.4 ± 0.5 | 6.1 ± 0.8 |  |
| 24 weeks | 6.3 ± 0.5 | 6.4 ± 0.7 |  | 6.1 ± 0.4 | 6.0 ± 0.5 |  |
| %Change | −1.0 ± 6.3 | −3.8 ± 7.7 | 0.565 | −5.9 ± 2.8 | −2.1 ± 6.7 | 0.065 |
| Insulin (μIU/mL) |  |  |  |  |  |  |
| Baseline | 22.4 ± 13.7 | 12.7 ± 6.4 |  | 11.8 ± 7.0 | 9.3 ± 4.2 |  |
| 24 weeks | 15.8 ± 7.7^†^ | 11.6 ± 7.7 |  | 11.9 ± 5.6 | 7.0 ± 4.6^⁎^ |  |
| %Change | −21.3 ± 20.5 | −1.4 ± 51.1 | 0.552 | 25.9 ± 64.3 | −21.7 ± 36.4 | 0.133 |
| HOMA-IR |  |  |  |  |  |  |
| Baseline | 7.2 ± 6.4 | 3.7 ± 2.0 |  | 3.6 ± 2.3 | 2.5 ± 1.3 |  |
| 24 weeks | 4.3 ± 2.2^⁎^ | 3.5 ± 2.6 |  | 3.6 ± 2.1 | 1.8 ± 1.1^⁎^ |  |
| %Change | −22.0 ± 29.1 | −0.5 ± 48.3 | 0.370 | 27.5 ± 71.3 | −25.6 ± 37.6 | 0.113 |

Variables are expressed as mean ± SD. ^⁎^P < 0.05; ^†^P < 0.01 vs. baseline value.

Period 1: Treatment period prior to washout; Period 2: Treatment period subsequent to washout. %Change, percentage change; HbA1c, hemoglobin A1c; and HOMA-IR, homeostasis model assessment of insulin resistance.

Supplemental TABLE 5 Changes in FMD values, baseline and maximal diameters of the brachial artery after pemafibrate or bezafibrate treatment during Period 1 and Period 2.

| Variable | Period 1 | |  | Period 2 | | P value (%Change) |
| --- | --- | --- | --- | --- | --- | --- |
|  | Pemafibrate  (n = 9) | Bezafibrate (n = 12) | P value (%Change) | Pemafibrate  (n = 12) | Bezafibrate (n = 9) |  |
| FMD (%) |  |  |  |  |  |  |
| Baseline | 5.51 ± 1.61 | 5.08 ± 1.57 |  | 5.87 ± 1.50 | 5.93 ± 1.84 |  |
| 24 weeks | 5.93 ± 2.95 | 6.09 ± 1.67^§^ |  | 6.41 ± 1.45^⁎^ | 6.83 ± 1.90^⁎^ |  |
| %Change | 11.4 ± 37.1 | 23.4 ± 18.4 | 0.165 | 15.4 ± 21.2 | 18.9 ± 24.8 | 0.694 |
| Baseline diameter (mm) |  |  |  |  |  |  |
| Baseline | 4.38 ± 0.65 | 4.33 ± 0.35 |  | 4.42 ± 0.45 | 4.07 ± 0.58 |  |
| 24 weeks | 4.31 ± 0.61 | 4.55 ± 0.34 |  | 4.67 ± 0.45 | 4.31 ± 0.59 |  |
| %Change | −1.3 ± 8.5 | 0.7 ± 11.3 | 0.373 | 1.2 ± 6.4 | 3.3 ± 9.5 | 0.548 |
| Maximal diameter (mm) |  |  |  |  |  |  |
| Baseline | 4.62 ± 0.66 | 4.34 ± 0.46 |  | 4.48 ± 0.58 | 4.18 ± 0.52 |  |
| 24 weeks | 4.56 ± 0.66 | 4.61 ± 0.48 |  | 4.76 ± 0.57 | 4.46 ± 0.53 |  |
| %Change | −1.6 ± 8.7 | 1.6 ± 11.5 | 0.330 | 1.9 ± 6.5 | 4.1 ± 8.9 | 0.494 |

Variables are expressed as mean ± SD. ^⁎^P < 0.05; ^§^P < 0.001 vs. baseline value.

Period 1: Treatment period prior to washout; Period 2: Treatment period subsequent to washout. %Change, percentage change; FMD, flow-mediated vasodilation.
